# Investigating the Effect of the Interaction of Maize Inducer and Donor Backgrounds on Haploid Induction Rates

**DOI:** 10.3390/plants11121527

**Published:** 2022-06-07

**Authors:** Henrique Uliana Trentin, Grigorii Batîru, Ursula Karoline Frei, Somak Dutta, Thomas Lübberstedt

**Affiliations:** 1 Bayer Crop Science, Coxilha 99145-000, RS, Brazil; henrique_trentin@hotmail.com; 2 Department of Agronomy and Environment, State Agrarian University of Moldova, 2049 Chisinau, Moldova; grigore.batiru@gmail.com; 3 Department of Agronomy, Iowa State University, Ames, IA 50011, USA; ufrei@iastate.edu; 4 Department of Statistics, Iowa State University, Ames, IA 50011, USA; somakd@iastate.edu

**Keywords:** maize, inducer background, donor background, haploid induction rate, haploid inducibility, doubled haploids, haploid seeds

## Abstract

Doubled haploid technology is a feasible, fast, and cost-efficient way of producing completely homozygous lines in maize. Many factors contribute to the success of this system including the haploid induction rate (HIR) of inducer lines, the inducibility of donor background, and environmental conditions. Sixteen inducer lines were tested on eight different genetic backgrounds of five categories in different environments for the HIR to determine possible interaction specificity. The HIR was assessed using the *R1-nj* phenotype and corrected using the red root marker or using a gold-standard test that uses plant traits. RWS and Mo-17-derived inducers showed higher average induction rates and the commercial dent hybrid background showed higher inducibility. In contrast, sweet corn and flint backgrounds had a relatively lower inducibility, while non-stiff stalk and stiff stalk backgrounds showed intermediate inducibility. For the poor-performing donors (sweet corn and flint), there was no difference in the HIR among the inducers. Anthocyanin inhibitor genes in such donors were assumed to have increased the misclassification rate in the F_1_ fraction and, hence, result in a lower HIR.

## 1. Introduction

Doubled haploid (DH) technology has become the main method of inbred line development in private and public maize breeding programs which possess the necessary infrastructure for adoption [1]. A significant reduction in time to produce highly homozygous lines, from 6 to 10 generations in recurrent selfing schemes [2] to 2 to 3 generations using the DH technique [3], explains the increasing adoption of this method. Additional advantages of this technique include simplified logistics, optimal exploitation of genetic variances in the testcross and per se levels, enhanced reproducibility of early selections and efficient gene stacking [4]. While maize DH lines can be produced both through tissue culture techniques (in vitro) and through genetic induction (in vivo), the former is often avoided due to its high cost and genotype dependency [4,5,6,7].

In vivo haploid induction in maize is made through intra-specific crosses with genotypes known as haploid inducers [5]. These genotypes have the intrinsic ability of generating seeds with haploid embryos in cross-pollination and can be used either as the pollen-source (male) or as the seed-parent (female) plant. When used as the pollen source, they are referred to as maternal inducers, since the genome of haploid embryos created is derived solely from the seed-parent plant. When used as the seed-parent plant, they are referred to as paternal inducers, since the nuclear genome of haploid embryos created is derived solely from the pollen-source plant. However, when inducers are used as the seed-parent plant, their cytoplasmic DNA is passed to their progeny. Since paternal inducers pass their cytoplasmic DNA to their progeny, they can be used to introduce cytoplasmic male sterility (CMS) to inbred lines [6,8]. For the creation of male-fertile DH lines, maternal inducers are preferred due to their higher haploid induction rates (HIRs). The HIR is calculated as the relative amount of seeds with haploid embryos over the total number of seeds produced in a cross pollination with a haploid inducer. While paternal inducers with a HIR of 6% have been developed [9], the HIR of maternal inducers was increased to more than 14% [4,10,11]. This difference impacts the number of crosses that need to be performed to obtain the desired number of seeds with haploid embryos and the time spent in their selection.

The differentiation between haploid and diploid seeds is largely performed visually, based on Purple Embryo Marker [12], which is encoded by the *R1-nj* gene. This gene leads to anthocyanin production in both the scutellum and aleurone layers of seeds, where proper fertilization and central cell development occurred. When inducer chromosomes are excluded from zygotic cells or the egg develops parthenogenetically, haploid embryos with unpigmented scutellum are formed. This difference in scutellum pigmentation allows the differentiation between haploid and diploid embryos [3].

Expression of *R1-nj* is affected by multiple factors, such as environmental conditions and donor genetic background [4,13,14,15]. Seed shape also influences scutellum visibility, being clearer in flat than in round seeds. Additionally, multiple alleles are known to inhibit *R1-nj* expression, such as c1 inhibitor (*c1-I*), c2 inhibitor diffuse (*c2-Idf*) and intensifier1 dominant (*in1-D*) [16,17,18]. Higher frequencies of these alleles in the flint, subtropical, tropical and sweet corn groups may explain the higher misclassification rates observed in these backgrounds [4,13,19,20].

The *Pl1* gene, which leads to light-independent, anthocyanin production in seedlings roots, was introgressed into some inducers because it serves as an additional mechanism for haploid and diploid differentiation. When present along with *B1*, *R1-r* or *r1-1*, *Pl-1* will also induce anthocyanin production in seedling coleoptiles, leaf tips, margins and sheaths [21]. Jointly, *B1* and *Pl-1* will also lead to a dark purple pigmentation on husks and culm [21]. Adult haploid and diploid plants differ in vigor, leaf erectness and male fertility, characteristics that are jointly analyzed in the gold-standard test of haploid and diploid discrimination [22]. Therefore, multiple pigmentation and morphological markers can be used to differentiate haploid and diploid plants in different phases of plant development. 

Environmental conditions, such as temperature and relative humidity, likely impact HIRs [4,6,13,23]. While Kebede et al. [13] observed higher HIRs in winter than in summer in Mexico, De La Fuente et al. [23] reported higher HIRs in a warmer than in a cooler Iowan summer. Silk age at the moment of pollination affects HIRs, with higher rates being observed in older silks [24,25,26,27]. Pollination method also impacts HIRs: hand-pollination leads to higher HIRs than open-pollination [28]. Heterofertilization was proposed as the cause of higher HIRs in hand-pollination [29,30,31]. 

The term inducibility is used to describe the impact that the donor parent has on HIRs [32]. Differences in the inducibility of source germplasm have long been reported [23,33,34,35,36], and their impact on the HIR can be very high. For instance, HIRs between 2.7% and 8.0% were observed when 20 different donors belonging to the flint, dent and flint × dent groups were pollinated by the same inducer [36]. De La Fuente et al. [23] observed a range of HIRs between 2.4% and 30.5% when 30 hybrids created out of a complete diallel of 6 inbred lines were pollinated by F_1_ or F_2_ plants of the haploid inducer RWS/RWK-76. Mean HIRs ranged from 0 to 11.3% using tropical donors [14]. 

Due to the complex influence that both inducer and donor parents have on HIRs, it is possible that there is an interaction between inducer and donor genetic backgrounds affecting the HIR. Highly significant genotypic differences were detected among inducers and source germplasm for HIRs in tropical conditions, but no interactions were observed between the two factors [14]. However, there is limited information for temperate maize. If this interaction is significant, then specific inducers should to be used to pollinate specific donors. Thus, the objectives of this research were to (i) compare the performance of inducers belonging to different genetic backgrounds, (ii) compare the inducibility of donors belonging to different genetic backgrounds and (iii) to determine if inducers belonging to different genetic backgrounds perform better on specific donor backgrounds.

## 2. Results

All factors considered had a highly significant effect (*p* < 0.001) on the HIR (Table 1). There was strong evidence for an interaction between inducer and donor background as well as for their main effects. Substantial year-to-year variation is also evidenced by a large F-ratio of 17.92 (the *p*-value not is reported because blocks are not randomly assigned to experimental units).

Both screeners (11.7) and residuals (8.2) explained the variance in the HIR more than any interactions between year, donor, and inducer (Table 2).

### 2.1. The Performance of Inducers Belonging to Different Genetic Backgrounds

RWS- and Mo-17-derived inducer backgrounds had higher average induction rates than the other inducers studied (Figure S1, Table 3 and Table S1). Overall, the HIR ranged from 5.02 to 8.02% among inducer backgrounds. Pairwise comparison grouped RWS- and Mo-17-derived inducers in one group (A), and A632.75/B15-derived, LH82-derived, LOR and PHI inducers in a separate group (B) with a significantly lower HIR. B73-derived inducers occupied an intermediate (AB) position with the two groups.

### 2.2. The Inducibility of Donors Belonging to Different Genetic Backgrounds

The commercial dent hybrid background showed a relatively higher inducibility (9.66) as compared to other donors (Table 4, Figure S2). In contrast, sweet corn and flint backgrounds showed lower inducibility—4.61 and 4.03, respectively—while non-stiff stalk and stiff stalk backgrounds showed intermediate inducibility. 

### 2.3. The Performance of Inducers Belonging to Different Genetic Backgrounds on Specific Donor Backgrounds

**Figure 1:**
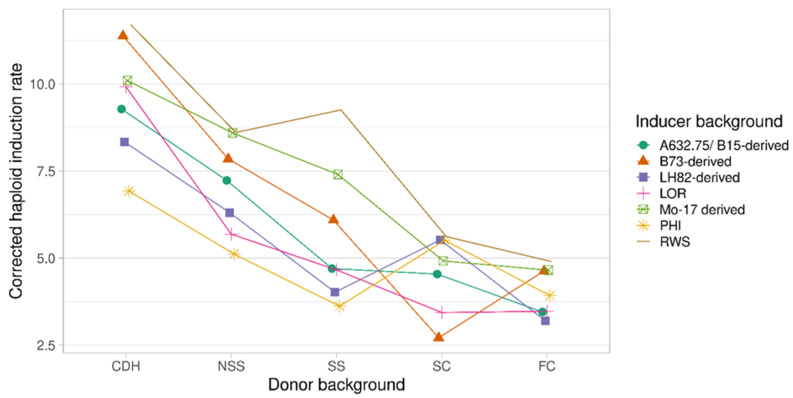
The interaction plot between the donor and inducer backgrounds. Abbreviations: CDH*—*commercial dent hybrid; NSS*—*non-stiff stalk; SS*—*stiff stalk; SC*—*sweet corn; FC*—*flint corn.

Our primary aim was to determine if inducers belonging to different genetic backgrounds perform better on specific donor backgrounds. From the data presented in Table 1, it was revealed that inducer by donor interactions were significant, though the effect was smaller than the main effects for inducer and for donor. To understand better the nature of these interactions for each donor background, we compared the HIR among inducer backgrounds. The interaction plot between donor and inducer backgrounds is shown in Figure 1 and Table S2.

The trend lines show that there were few changes in ranking for different backgrounds, with the exception of sweet corn, for most inducers. Overall, the RWS inducer was superior. The PHI inducer performed better in sweet corn background, but performed the worst for the commercial dent hybrid, stiff stalk and non-stiff stalk donors. The B73-derived inducers had the highest interaction with donors performing second in commercial dent hybrid, third in non-stiff stalk and stiff stalk, highest in the flint donor and lowest in sweet corn. However, inducers did not differ statistically for the two poor-performing donors (sweet corn and flint) (Table S3).

## 3. Discussion

DH technology consists of generating haploid seeds from crosses of inducer lines with donors of interest followed by selection of haploids based on the *R1-nj* phenotype. This system is highly dependent on many factors including inducer lines, the inducibility of donor background, and environmental conditions [37]. In our experiment, all factors had a significant effect on the HIR, which confirms that in vivo haploid induction is influenced by both genetic and non-genetic variation. The HIR showed variability both among inducers and donors, demonstrating the quantitative nature of the induction ability of inducers and the inducibility of donors. Generally, superior environments and optimizing the growing conditions of the donor and inducer plants increase the induction rate [4,6].

However, the flint and sweet corn backgrounds showed overall lower HIRs compared to dent genotypes. Selection of haploid seeds in such backgrounds is hampered due to variation in *R1-nj* expression, leading to a high misclassification rate. Presence of dominant anthocyanin inhibitor genes such as *C1-I*, *C2-Idf*, and *In1-D* in donor backgrounds or dosage effects can make this marker ineffective in haploid selection [21]. If dominant anthocyanin inhibitor genes such as *C1-I*, which are common in flint maize, are present, *R1-nj* color marker expression is completely suppressed and haploid seed identification is almost impossible [4]. Large variations in the Navajo phenotype and inhibition of *R1-nj* expression were observed in the majority of crosses between inducers and commercial sweet corn hybrids [20]. When F_1_ or F_2_ populations are used as source materials and when only one parent has inhibitor genes, seeds will segregate for the Navajo phenotype. In such cases, one may not be able to identify all haploid seeds efficiently and could potentially lose half to three-quarters of the haploids [4].

According to Prigge et al. [14], there are two types of incorrect decisions in haploid identification systems: haploid seeds or plants are discarded by mistake (type I error), i.e., false positives; or normal F_1_ seeds or plants are misclassified as haploids (type II error), i.e., false negatives, given that the null hypothesis (H_0_) assumes that the seeds are haploids. A type I error may occur due to the limited efficacy of the *R1-nj* color marker or due to insufficiently trained technical staff. In our case, screeners caused the most variation (Table 2). In experiments with dent and flint maize [4], the average proportion of verified haploids within the putative haploid fraction amounted to 89.6% in the dent and only 48.0% in the flint group. Many flint genotypes displayed a similarly strong marker expression to the dent group. Flint samples with a low proportion of verified haploids tended towards a high percentage of undetected haploids in the putative F_1_ fraction [4]. In our experiments, the average proportion of verified haploids within the putative haploid fraction amounted to 88.5% in the commercial dent donor, 62.8% in non-stiff stalk, 41.0% in stiff stalk, 16.3% in the flint group and only 13.7% in sweet corn. The HIR was corrected for a type II error and not corrected for a type I error. This may explain the low induction rate in flint and sweet corn as compared to dent types in our experiment. These donors have a high rate of misclassification as noted above. However, a relatively high number of putative haploid seeds selected in these donors might have reduced the type I error to a type II error that was corrected by means of an independent *Pl1*-mediated red root marker and gold-standard tests.

Some authors [14] suggest that screening seeds for haploidy at the time of harvesting or before drying may reduce the occurrence of a type I error because *R1-nj*-encoded embryo coloration is usually more clearly visible at this stage. In contrast, if seeds are screened after drying, true haploids may be inadvertently discarded. During drying, sometimes air pockets develop underneath the pericarp region covering the embryos, which causes the appearance of darker shades that may be incorrectly perceived as embryo pigmentation. Similarly, seeds carrying a haploid embryo but exhibiting very poor endosperm coloration may be misclassified as non-pigmented seeds [14].

In previous studies [4], *R1-nj* color expression was inhibited in only approximately 8% of crosses of haploid inducers with diverse source populations. Complete color inhibition was revealed in ~4% of entries in tropical breeding populations, ~27% in the landraces, and ~30% in inbred lines [19].

It was suggested [4] that for flint and some dent donors, inhibitor genes have to be eliminated before the *R1-nj* marker can efficiently be used in breeding programs. Screening for color inhibition is easy due to a simple, mostly monogenic inheritance of this trait and can readily be combined with the routine DH line development. The intensity of the scutellum and aleurone coloration in donors without inhibitor gene(s) is similar in dent and flint materials [4]. Eder and Chalyk [36] found an even more intense scutellum pigmentation in flint than in dent or flint × dent donors.

The interaction plot suggests that the RWS inducer performed better than other inducers in all donors considered in this study. In commercial dent background, most of the inducers bar PHI had a higher HIR than in other backgrounds. For non-stiff stalk backgrounds, RWS, Mo17-derived, B73-derived and A632.75/B15-derived inducers were equally efficient; for stiff stalk backgrounds, only RWS and Mo17-derived inducers showed better performance (Table S3). The data in Figure 1 suggest that the PHI inducer can also be used in the sweet corn background. However, there was no statistical difference in performance between inducer backgrounds in sweet and flint corn. While the selected donors are typical representatives of germplasm groups, and some inducers sustain an advantage over others in terms of the HIR, more extensive studies with more donors are needed to identify the best-matched inducers for a better recommendation. The decrease in the HIR from commercial hybrids to flint corn in Figure 1 is actually accompanied by an increase in the difficulty of haploid selection. With more advanced haploid discrimination methods, it should be possible to make more accurate decisions on which inducers to use in donor germplasms to maximize HIRs in a given maize germplasm.

## 4. Materials and Methods

### 4.1. Plant Materials

The sixteen inducers used in this experiment can be grouped into seven distinct backgrounds based on their pedigree. Twelve of the sixteen inducers were developed by the DH Facility of Iowa State University (DHF-ISU), and have inducers RWS and RWK-76 as their source of haploid induction ability. BHI305, BHI306 and BHI307 are near isogenic inducers developed from the inbreds A632.75 and B-15 dent sterile (A632.75/B-15-derived). BHI201, BHI101 and BHI103 are near isogenic inducers developed in from the inbred B73 (B73-derived). Three near isogenic inducers derived from the inbred Mo17 do not have commercial names, and are referred to here as Mo-15, Mo-17, and Mo-23 (Mo17-derived). Three near isogenic inducers derived from the inbred LH82 also have no commercial names, and are referred to as LH82-26, LH82-28 and LH82-29 (LH82-derived). The following inducers were developed in Europe. RWS was developed at the University of Hohenheim, by crossing inducers WS14 and KEMS [4]. Inducer PHI-3 was developed by Procera Agrochemicals (Fundulea, Romania), by crossing inducer MHI with Stock 6 [10]. Inducers LOR3758 and LOR3759 were developed by MAS Seeds (Haut-Mauco, France) and their background is unknown (LOR).

The eight donors used in this experiment can be divided into five different genetic groups. Viking 60-01N is a commercial dent corn hybrid developed by Albert Lea Seed and is presumably derived from a cross of two inbred lines belonging to different heterotic groups. Golden Jubilee is a commercial sweet corn hybrid also developed by Albert Lea Seed (Albert Lea, MN, USA). Two F_1_ hybrids were created within the Lancaster (non-stiff stalk) heterotic group by crossing the inbreds PHN82 with PHP76, and PHG29 with PHG83. Two F_1_ hybrids were created within the stiff stalk synthetic heterotic group, by crossing the inbreds PHG86 with PHW17, and LH206 with PHW52. Two F_1_ flint hybrids, LFN1971.LGR2038 and LFR1941.19944, belonging to MAS Seeds, were used to represent the flint group. The reason for using hybrids rather than inbreds was due to their higher seed set, which has an impact on the power of the statistical analyses.

### 4.2. Experimental Design

This experiment was conducted during the summers of 2016, 2017 and 2018 at the Iowa State University Agricultural Engineering and Agronomy Farm, located in Boone, Iowa. To ensure nicking, during the summers of 2016 and 2017, inducers were planted on a single planting date, while donors were planted on two distinct planting dates. Pollination was preferably performed on donor plants that presented fresh silks, which were trimmed to a size of 2 cm before pollination. During the summer of 2018, inducers and donors were planted on two planting dates, with the earliest planting of inducers used to pollinate the earliest planting of donors, and the latest planting of inducers for the latest planting of donors. During the summers of 2016 and 2017, inducers and donors were planted in 3.8 m-long plots. Each inducer had one planting with four rows and each donor had two plantings with two rows with twenty seeds per row. During the summer of 2018, each inducer and donor was planted in one 5.4 m-long plot, on two planting dates, with twenty-five seeds per row. Row spacing was of 0.76 m during all years. All trials were grown on loam soils, under rainfed conditions and adopting standard agronomic practices for maize production in Iowa. Pre-emergent herbicides and hoeing were used for weed control.

All donor plants were manually detasseled and shoots were covered using glassine bags before silk exposure. Bulk pollen of each inducer was collected in brown tassel bags and used to pollinate at least ten ears of each donor genotype. Each donor plot was properly labeled and pollinated by a single inducer. All pollinated ears from a plot were bulk harvested when seeds reached the harvest maturity stage.

### 4.3. Phenotypic Evaluation

The ploidy level of the embryo of each seed was evaluated using the *R1-nj* marker [19]. Each ear constituted a different experimental unit; and for each ear, the number of seeds with putative haploids and diploid embryos were recorded. Putative haploid seeds of each inducer by donor combination were bulked, and a sample bigger than 200 seeds was planted to correct for misclassification rates. The inducers BHI305, BHI306, BHI307 and PHI-3 carry the *Pl-1* allele, which leads to anthocyanin production on seedling roots. Putative haploids generated in crosses with these inducers were planted in the greenhouse and verification was performed using the root color marker. For all other inducers, putative haploids were planted in the field and their ploidy verified using the gold-standard test based on visual assessment of differences in plant vigor, erectness of leaves, and male fertility in haploids and diploids [22]. These putative haploid plants were grown under the same conditions and following the same practices as the inducer and donor plants.

The HIR of each ear was corrected by multiplying the calculated HIR with the frequency of true haploid plants observed using the red root marker or based on the gold-standard test [13,22]. Data from ears where seed set was under 20 were filtered out, since seeds tend to grow larger and, as a consequence, harder to discriminate. Data from ears where the HIR was above 25% were also eliminated, since these induction rates are not expected for the inducers used in this experiment. Screeners were instructed to classify all seeds where the ploidy level could not be easily determined as being putative haploids.

### 4.4. Statistical Analysis

The statistical model used for the analysis is described below:

Y*_ijklmn_* = μ + b*_i_* + s*_j_* + α*_k_* + β*_l_* + (αβ)*_kl_* + g*_ikm_* + g′*_iln_* + g″*_iklmn_* + ϵ*_ijklmn_* (1)

where Y*_ijklmn_* is the HIR on the *i*th year, by the *j*th screener, from the *m*th inducer from the *k*th inducer background and the *n*th donor from the *l*th donor background; μ is an overall effect, b*_i_* is the fixed block (i.e., year) effect, s*_j_* is the random screener effect, α*_k_* is the inducer background main effect, β*_l_* is the donor background main effect, and (αβ)*_kl_* is the interaction effect. g*_ikm_* is the random inducer effect (nested within inducer background), g′*_iln_* is the random donor effect (nested within the donor background), g″*_iklmn_* is the random interaction effect between the donor and the inducer (nested within their respective backgrounds), and ϵ*_ijklmn_* is the residual error.

Following model fit, the marginal means of inducer backgrounds, donor backgrounds, and their combinations were obtained and Tukey’s adjusted pairwise comparisons were performed. Pairwise comparisons were summarized by connected letters reports, pairwise *p*-value plots, and pairwise *p*-value matrices. Statistical analyses and visualization were conducted using R [38].

## 5. Conclusions

Inducer and donor backgrounds considered in this experiment had a significant effect (*p* < 0.001) on the HIR. RWS and Mo-17-derived inducer backgrounds had higher average induction rates than the other inducers studied. The commercial dent hybrid was determined as a donor with high inducibility. Sweet corn and flint corn showed low inducibility, with no statistical difference between the inducers. Non-stiff stalk and stiff stalk backgrounds showed intermediate inducibility. More extensive studies with more donors and germplasm groups are needed to identify the best-matched inducers. Anthocyanin inhibitor genes in poor-performing donors were assumed to have increased the misclassification rate in the F_1_ fraction, which led to a lower HIR.

## Figures and Tables

**Table 1:** The ANOVA table of fixed effects tested for the HIR.

Factor	SS	MS	Num DF	Den DF	F Value	Pr (>F)
Year	439.8	146.6	3	36.4	17.92	
Inducer background	382.3	63.7	6	68.2	7.79	<0.001
Donor background	375.1	93.8	4	24.0	11.46	<0.001
Inducer background × donor background	421.4	17.6	24	336.9	2.15	<0.001

SS*—*sum of squares, MS*—*mean squares, Num DF*—*numerator degrees of freedom, and Den DF*—*denominator degrees of freedom.

**Table 2:** Factors contributing to variance in the HIR.

Groups	Variance
Screener	11.650
Year × Inducer	0.265
Year × Donor	1.777
Year × Donor × Inducer	3.102
Residual	8.179

**Table 3:** Pairwise comparisons of the mean HIR in inducer backgrounds.

Inducer Background	Mean (%)	SE	Asymp. LCL	Asymp. UCL	Group
RWS	8.02	0.780	6.49	9.55	A
Mo17-derived	7.13	0.675	5.81	8.46	A
B73-derived	6.53	0.727	5.11	7.96	AB
A632.75/B15-derived	5.84	0.676	4.52	7.17	B
LH82-derived	5.48	0.676	4.15	6.80	B
LOR	5.44	0.710	4.04	6.83	B
PHI	5.02	0.781	3.49	6.55	B

SE*—*standard error; LCL*—*lower control limit; UCL*—*upper control limit. Means in the same group are not significantly different.

**Table 4:** Pairwise comparisons of the mean HIR in donor backgrounds.

Donor Background	Mean (%)	SE	Asymp. LCL	Asymp. UCL	Group
Commercial dent hybrid	9.66	0.923	7.85	11.47	A
Non-stiff stalk	7.06	0.767	5.55	8.56	B
Stiff stalk	5.68	0.766	4.18	7.18	BC
Sweet corn	4.61	0.948	2.75	6.47	BC
Flint corn	4.03	0.795	2.47	5.59	C

SE*—*standard error; LCL*—*lower control limit; UCL*—*upper control limit. Means in the same group are not significantly different.

## Data Availability

Data are available within this article or the Supplementary Materials.

## Supplementary Materials

The following supporting information can be downloaded at: https://www.mdpi.com/article/10.3390/plants11121527/s1, Figure S1: Pairwise *p*-value plot for the haploid induction rate; Table S1: Pairwise comparison matrix for the haploid induction rate; Figure S2: Pairwise *p*-value plot for inducibility; Table S2: Estimated marginal means (lsmeans) of the performance of inducer backgrounds on specific donor backgrounds; Table S3: Pairwise comparisons of inducer backgrounds within each donor background.
