# Supplementary material for: Investigating the Effect of the Interaction of Maize Inducer and Donor Backgrounds on Haploid Induction Rates
Source: Plants (Basel). 2022 Jun 7;11(12):1527. doi: 10.3390/plants11121527 (PMC9228012; doi:10.3390/plants11121527)
Supplement: Supplementary file 1 [file plants-11-01527-s001.zip › plants-1743032-supplementary.pdf]

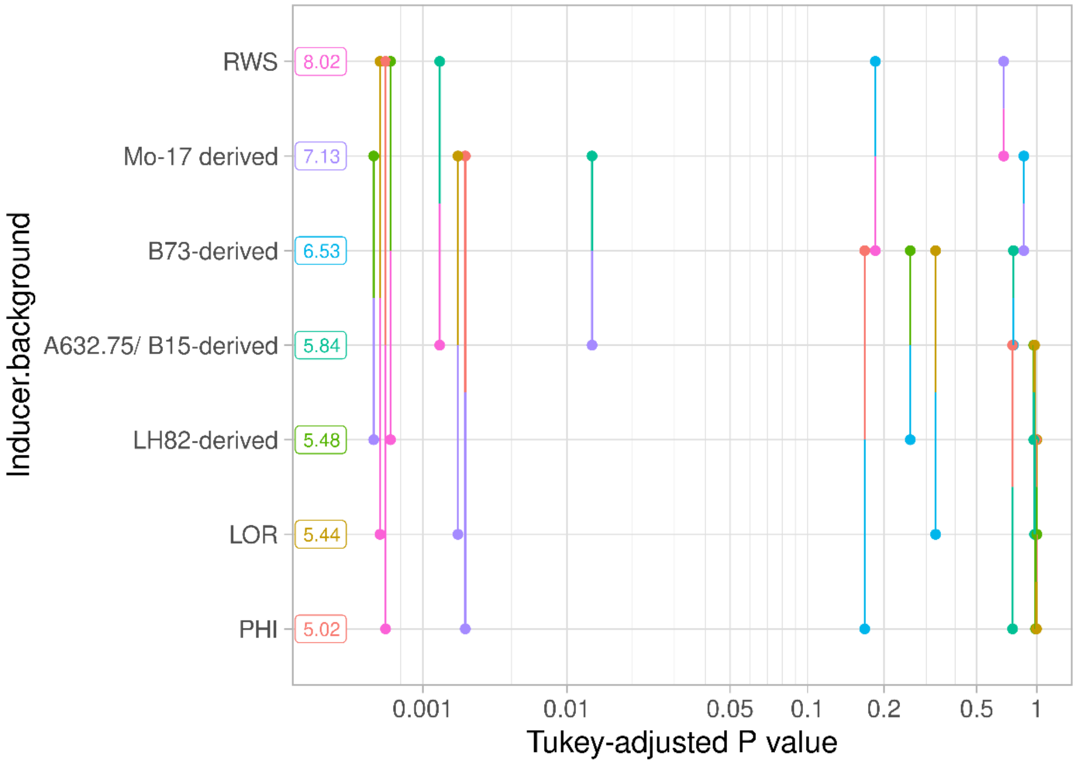

Figure S1. The pairwise p-value plot for Haploid Induction Rate.

Table S1. Pairwise comparison matrix for HIR.

Inducer background	A632.75/ B15-derived	B73-derived	LH82-derived	LOR	Mo-17 derived	PHI	RWS
A632.75/B15-derived	[5.84]	0.7528	0.9642	0.9694	0.0132	0.7505	0.0015
B73-derived	-0.6910	[6.53]	0.2557	0.3278	0.8522	0.1674	0.1844
LH82-derived	0.3638	1.0548	[5.48]	1.0000	0.0003	0.9815	<.0001
LOR	0.4050	1.0959	0.0411	[5.41]	0.0021	0.9923	0.0002
Mo17-derived	-1.2927	-0.6018	-1.6566	-1.6977	[7.13]	0.0025	0.6767
PHI	0.8220	1.5130	0.4582	0.4171	2.1148	[5.02]	0.0002
RWS	-2.1780	-1.4870	-2.5418	-2.5829	-0.8852	-3.0000	[8.02]

The diagonals show the marginal means, lower triangle shows the pairwise difference estimates and the upper triangle shows the Tukey-adjusted p-values of the pairwise comparisons.

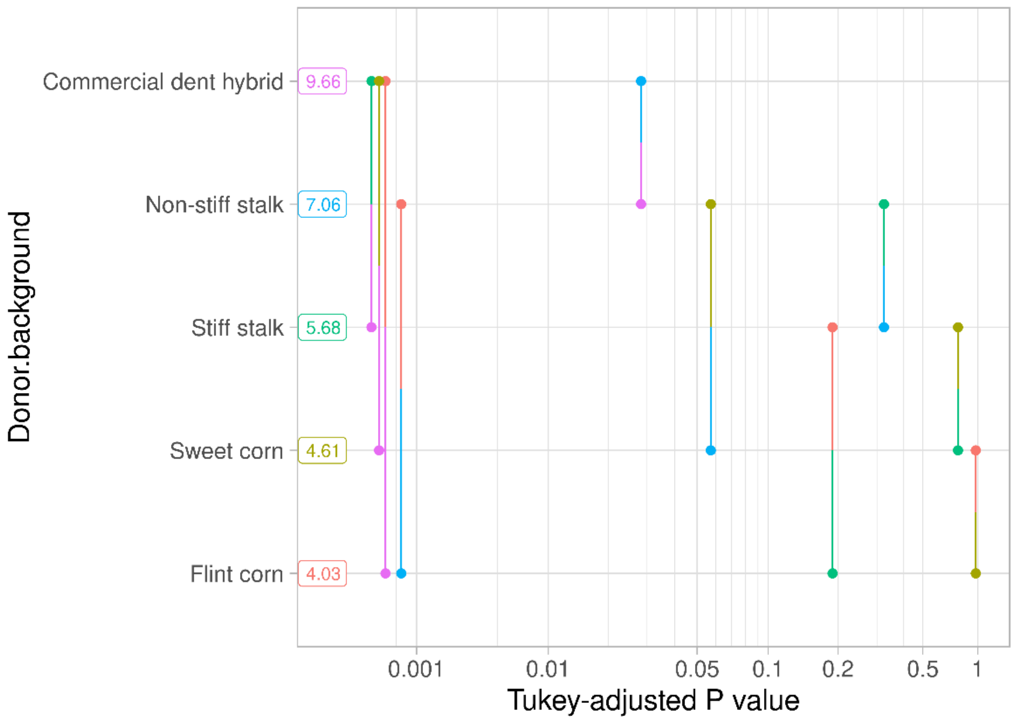

Figure S2. The pairwise p-value plot for inducibility of donors belonging to different genetic backgrounds.

Table S2. The estimated marginal means (*lsmeans*) of performance of inducer backgrounds on specific donor backgrounds.

##	Inducer background	Donor background	emmean	SE	asymp.LCL	asymp.UCL
1	A632.75/ B15-derived	Commercial dent hybrid	9.28	1.055	7.2134	11.35
2	B73-derived		11.38	1.113	9.1979	13.56
3	LH82-derived		8.34	1.055	6.2666	10.40
4	LOR		9.92	1.142	7.6807	12.16
5	Mo17-derived		10.10	1.058	8.0228	12.17
6	PHI		6.92	1.360	4.2576	9.59
7	RWS		11.71	1.336	9.0912	14.33
8	A632.75/ B15-derived	Non-Stiff stalk	7.23	0.856	5.5535	8.91
9	B73-derived		7.85	0.920	6.0436	9.65
10	LH82-derived		6.31	0.853	4.6320	7.98
11	LOR		5.69	0.924	3.8771	7.50
12	Mo-17 derived		8.59	0.860	6.9081	10.28
13	PHI		5.11	1.051	3.0547	7.17
14	RWS		8.61	1.049	6.5512	10.66
15	A632.75/ B15-derived	Stiff Stalk	4.70	0.861	3.0118	6.39
16	B73-derived		6.09	0.869	4.3879	7.80
17	LH82-derived		4.02	0.856	2.3425	5.70
18	LOR		4.66	0.918	2.8626	6.46
19	Mo17-derived		7.40	0.858	5.7195	9.08
20	PHI		3.62	1.068	1.5260	5.71
21	RWS		9.26	1.062	7.1760	11.34
22	A632.75/ B15-derived	Flint corn	3.45	0.898	1.6919	5.21
23	B73-derived		4.63	1.204	2.2665	6.99

24	LH82-derived		3.20	0.884	1.4659	4.93
25	LOR		3.47	1.000	1.5134	5.43
26	Mo17-derived		4.65	0.894	2.9009	6.40
27	PHI		3.92	1.169	1.6307	6.21
28	RWS		4.90	1.112	2.7230	7.08
29	A632.75/ B15-derived	Sweet Corn	4.54	1.097	2.3907	6.69
30	B73-derived		2.71	1.424	-0.0788	5.50
31	LH82-derived		5.52	1.104	3.3597	7.69
32	LOR		3.44	1.241	1.0043	5.87
33	Mo17-derived		4.92	1.081	2.8019	7.04
34	PHI		5.51	1.446	2.6787	8.35
35	RWS		5.62	1.497	2.6832	8.55

Table S3. Pairwise comparisons of the inducer backgrounds within each donor background.

Donor background = *Commercial dent hybrid*:

Inducer background	emmean	SE	df	Asymp. LCL	Asymp. UCL	group
RWS	11.71	1.336	Inf	9.0912	14.33	AB
B73-derived	11.38	1.113	Inf	9.1979	13.56	A
Mo17-derived	10.10	1.058	Inf	8.0228	12.17	ABC
LOR	9.92	1.142	Inf	7.6807	12.16	ABC
A632.75/B15-derived	9.28	1.055	Inf	7.2134	11.35	ABC
LH82-derived	8.34	1.055	Inf	6.2666	10.40	BC
PHI	6.92	1.360	Inf	4.2576	9.59	C

Donor background = *Non-stiff stalk*

Inducer background	emmean	SE	df	Asymp. LCL	Asymp. UCL	group
RWS	8.61	1.049	Inf	6.5512	10.66	AB
Mo17-derived	8.59	0.860	Inf	6.9081	10.28	A
B73-derived	7.85	0.920	Inf	6.0436	9.65	ABC
A632.75/B15-derived	7.23	0.856	Inf	5.5535	8.91	ABC
LH82-derived	6.31	0.853	Inf	4.6328	7.98	BC
LOR	5.69	0.924	Inf	3.8771	7.50	C
PHI	5.11	1.051	Inf	3.0547	7.17	C

Donor background = *Stiff stalk*:

Inducer background	emmean	SE	df	Asymp. LCL	Asymp. UCL	group
RWS	9.26	1.062	Inf	7.1760	11.34	A
Mo17-derived	7.40	0.858	Inf	5.7195	9.08	AB
B73-derived	6.09	0.869	Inf	4.3879	7.80	BC
A632.75/B15-derived	4.70	0.861	Inf	3.0118	6.39	CD
LOR	4.66	0.918	Inf	2.8626	6.46	CD
LH82-derived	4.02	0.856	Inf	2.3425	5.70	D
PHI	3.62	1.068	Inf	1.5260	5.71	CD

Donor background = *Flint Corn*

Inducer background	emmean	SE	df	Asymp. LCL	Asymp. UCL	group
RWS	4.90	1.112	Inf	2.7230	7.08	A
Mo17-derived	4.65	0.894	Inf	2.9009	6.40	A

B73-derived	4.63	1.204	Inf	2.5665	6.99	A
PHI	3.92	1.169	Inf	1.6307	6.21	A
LOR	3.47	1.000	Inf	1.5134	5.43	A
A632.75/B15-derived	3.45	0.989	Inf	1.6919	5.21	A
LH82-derived	3.20	0.884	Inf	1.4659	4.93	A

Donor background = *Sweet Corn*:

Inducer background	emmean	SE	df	Asymp. LCL	Asymp. UCL	group
RWS	5.62	1.497	Inf	2.6832	8.55	A
LH82-derived	5.52	1.104	Inf	3.3597	7.69	A
PHI	5.51	1.446	Inf	2.6787	8.35	A
Mo17-derived	4.92	1.081	Inf	2.8019	7.04	A
A632.75/B15-derived	4.54	1.097	Inf	2.3907	6.69	A
LOR	3.44	1.241	Inf	1.0043	5.87	A
B73-derived	2.71	2.424	Inf	-0.0788	5.50	A
